# Comparative molecular analyses of left-sided colon, right-sided colon, and rectal cancers

**DOI:** 10.18632/oncotarget.21169

**Published:** 2017-09-21

**Authors:** Mohamed E. Salem, Benjamin A. Weinberg, Joanne Xiu, Wafik S. El-Deiry, Jimmy J. Hwang, Zoran Gatalica, Philip A. Philip, Anthony F. Shields, Heinz-Josef Lenz, John L. Marshall

**Affiliations:** ^1^ Ruesch Center for The Cure of Gastrointestinal Cancers, Lombardi Comprehensive Cancer Center, Georgetown University Medical Center, Washington, DC, USA; ^2^ Caris Life Sciences, Phoenix, AZ, USA; ^3^ Fox Chase Cancer Center, Philadelphia, PA, USA; ^4^ Levine Cancer Institute, Carolinas HealthCare System, Charlotte, NC, USA; ^5^ Department of Oncology, Karmanos Cancer Institute, Wayne State University, Detroit, MI, USA; ^6^ Norris Comprehensive Cancer Center, University of Southern California, Los Angeles, CA, USA

**Keywords:** colorectal neoplasms, colonic neoplasms, rectal neoplasms, DNA mutational analysis, immunohistochemistry

## Abstract

Tumor sidedness has emerged as an important prognostic and predictive factor in the treatment of colorectal cancer. Recent studies demonstrate that patients with advanced right-sided colon cancers have a worse prognosis than those with left-sided colon or rectal cancers, and these patient subgroups respond differently to biological therapies. Historically, management of patients with metastatic colon and rectal cancers has been similar, and colon and rectal cancer patients have been grouped together in large clinical trials. Clearly, the differences in molecular biology among right-sided colon, left-sided colon, and rectal cancers should be further studied in order to account for disparities in clinical outcomes. We profiled 10,570 colorectal tumors (of which 2,413 were identified as arising from the left colon, right colon, or rectum) using next-generation sequencing, immunohistochemistry, chromogenic in-situ hybridization, and fragment analysis (Caris Life Sciences, Phoenix, AZ). Right-sided colon cancers had higher rates of microsatellite instability, more frequent aberrant activation of the EGFR pathway including higher *BRAF* and *PIK3CA* mutation rates, and increased mutational burden compared to left-sided colon and rectal cancers. Rectal cancers had higher rates of TOPO1 expression and Her2/neu amplification compared to both left- and right-sided colon cancers. Molecular variations among right-sided colon, left-sided colon, and rectal tumors may contribute to differences in clinical behavior. The site of tumor origin (left colon, right colon, or rectum) should certainly be considered when selecting treatment regimens and stratifying patients for future clinical trials.

## INTRODUCTION

The impact of tumor location on patient survival and response to therapies has been shown in large clinical trials; however, the underlying tumor biology explaining these differences has not been systematically explored. In fact, cancers arising from the colon and rectum are often grouped together and generally categorized as colorectal cancer (CRC) despite their distinctly different clinical behaviors and management needs. The right colon has a different embryological origin and blood supply from the left colon and rectum. The superior mesenteric artery supplies midgut structures from the mid-duodenum to the mid-transverse colon, whereas the inferior mesenteric artery supplies hindgut structures from the mid-transverse colon to the rectum [1]. CRC has recently been divided into four consensus molecular subtypes (CMS): microsatellite instability immune (CMS1), canonical (CMS2), metabolic (CMS3), and mesenchymal (CMS4) [2]. The differential distribution of the four classes in various anatomic regions suggests biological differences in right-sided colon, left-sided colon, and rectal tumors. We investigated the proteomic and genetic aberrations of a large cohort of clinical CRC samples to further delineate these molecular differences.

A potentially practice-changing retrospective analysis of the pivotal CALGB/SWOG 80405 study presented at the 2016 ASCO Annual Meeting demonstrated that patients with *KRAS* wild-type metastatic colon cancer from a right-sided primary colon tumor had shorter median overall survival (mOS, 19.4 months) than patients with metastatic left-sided primary colon cancers (33.3 months, hazard ratio [HR] = 1.55, 95% confidence interval [CI] = 1.32-1.82, P <.0001) [3]. Among patients who received cetuximab, mOS was 36 months for patients with left-sided tumors but only 16.7 months for patients with right-sided tumors (HR = 1.87, 95% CI = 1.48-2.32, P <.0001). Following bevacizumab treatment, mOS was 31.4 months for patients with left-sided tumors and 24.2 months for those with right-sided tumors (HR = 1.32, 95% CI = 1.05-1.65, P = 0.01). Moreover, bevacizumab led to better outcomes than cetuximab among patients with right-sided tumors regardless of *KRAS* mutational status, whereas cetuximab performed better than bevacizumab in patients with *KRAS* wild-type left-sided tumors. Similarly, the Canadian NCIC CO.17 trial of 399 patients showed that the OS benefit of cetuximab was more pronounced in patients with left-sided tumors, regardless of *KRAS* mutational status [4]. Coincidentally, the recent retrospective analysis of the FIRE-3 and CRYSTAL trials inarguably showed that patients with left-sided tumors had a markedly better prognosis than those with right-sided tumors and that first-line FOLFIRI plus cetuximab clearly benefited patients with left-sided tumors whereas patients with right-sided tumors derived limited benefit from any standard treatment [5].

An analysis of the SEER database showed that patients with right-sided stage III or IV colon cancers had inferior mOS when compared to left-sided colon and rectal cancers [6]. Another study profiled 198 *KRAS* wild-type metastatic CRCs (mCRC) and found that right-sided tumors were associated with having a high CpG island methylator phenotype (CIMP, odds ratio [OR] = 2.35, 95% CI 1.22-4.54) and being *BRAF* mutation-positive (OR = 5.45, 95% CI 2.47-12.03), conferring worse survival outcomes and a poorer response to anti-epidermal growth factor receptor (EGFR) therapies [7]. Thus, it appears that metastatic tumors arising in the left versus right side of the colon are biologically different and respond differently to therapy.

We examined 2,413 biospecimens to determine whether primary colon and rectal cancers are molecularly different. If there are biological differences, these should be defined due to their potential to affect tumor response to chemotherapy, targeted biological therapy, or immunotherapy. Furthermore, it is quite possible that future CRC clinical trials should incorporate stratification of patients according to their primary site of disease.

## RESULTS

### Tumor characteristics

**Figure 1:**
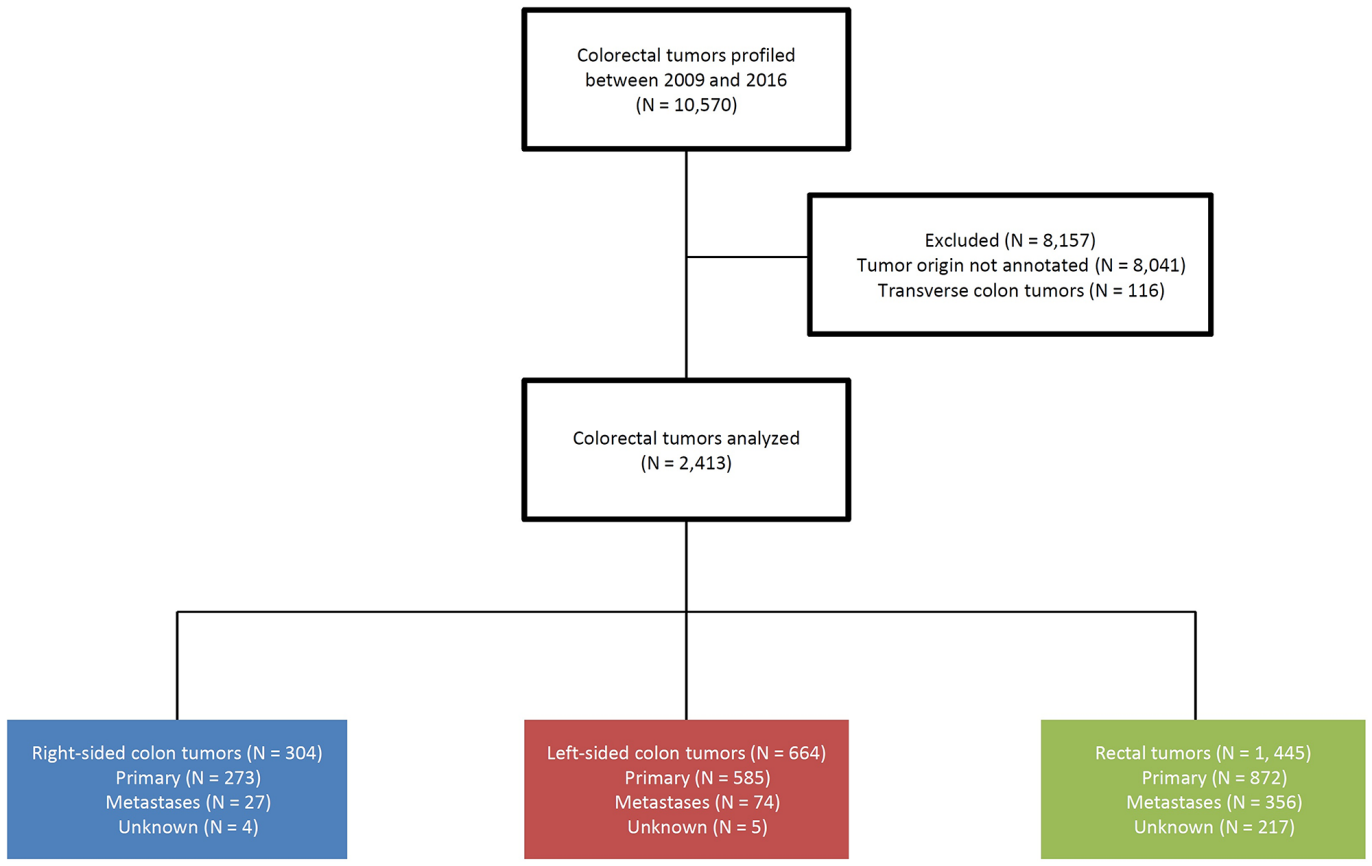
Diagram of colorectal tumors included in this study

Profiling of 10,570 colorectal tumors took place, but only 2,529 of these had clearly annotated tumor origins. Transverse colon tumors (116) were excluded, and our analysis included 304 right colon tumors, 664 left colon tumors, and 1,445 rectal tumors (Figure 1). A total of 1,730 samples were from primary tumors, 457 were from metastases, and 226 tumors could not be identified as primary or metastatic. The lower than expected number of colon cancers was due to the exclusion of many cases that did not specify the location within the colon. Biomarker comparisons were performed on the 1,730 primary tumors, including 1,424 tested with immunohistochemistry (IHC), 753 with next-generation sequencing (NGS) on the MiSeq platform, and 70 with NGS on the NextSeq platform.

### Patient characteristics

Patients with right-sided colon tumors were significantly older than patients with left-sided colon tumors (64 vs. 59 years, P < 0.001) as well as those with rectal tumors (64 vs. 59 years, P < 0.001). The age of patients with left-sided colon tumors was similar to that of patients with rectal tumors (P = 0.79). Sixty-two percent of rectal tumors were from male patients, and this was significantly higher than the male percentage of left- (54%, P = 0.001) and right-sided (44%, P < 0.0001) colon tumors (Table 1).

**Table 1:** Patient characteristics of age and gender by tumor location (right colon, left colon, or rectal)

	Right colon	Left colon	Rectum
**Age** (median, interquartile range in years)	64 (54-74)	59 (50-68)	59 (51-67)
	Right vs. Left: P = 6.2E-08, Left vs. Rectum: P = 0.79, Right vs. Rectum: P = 1.0E-08		
**Gender** (% male)	44%	54%	62%
	Right vs. Left: P = 0.0046, Left vs. Rectum: P = 0.001, Right vs. Rectum: P < 0.0001		

### Comparison of molecular alterations of 1,730 primary tumors based on their site of origin

#### Common CRC mutations

**Figure 2:**
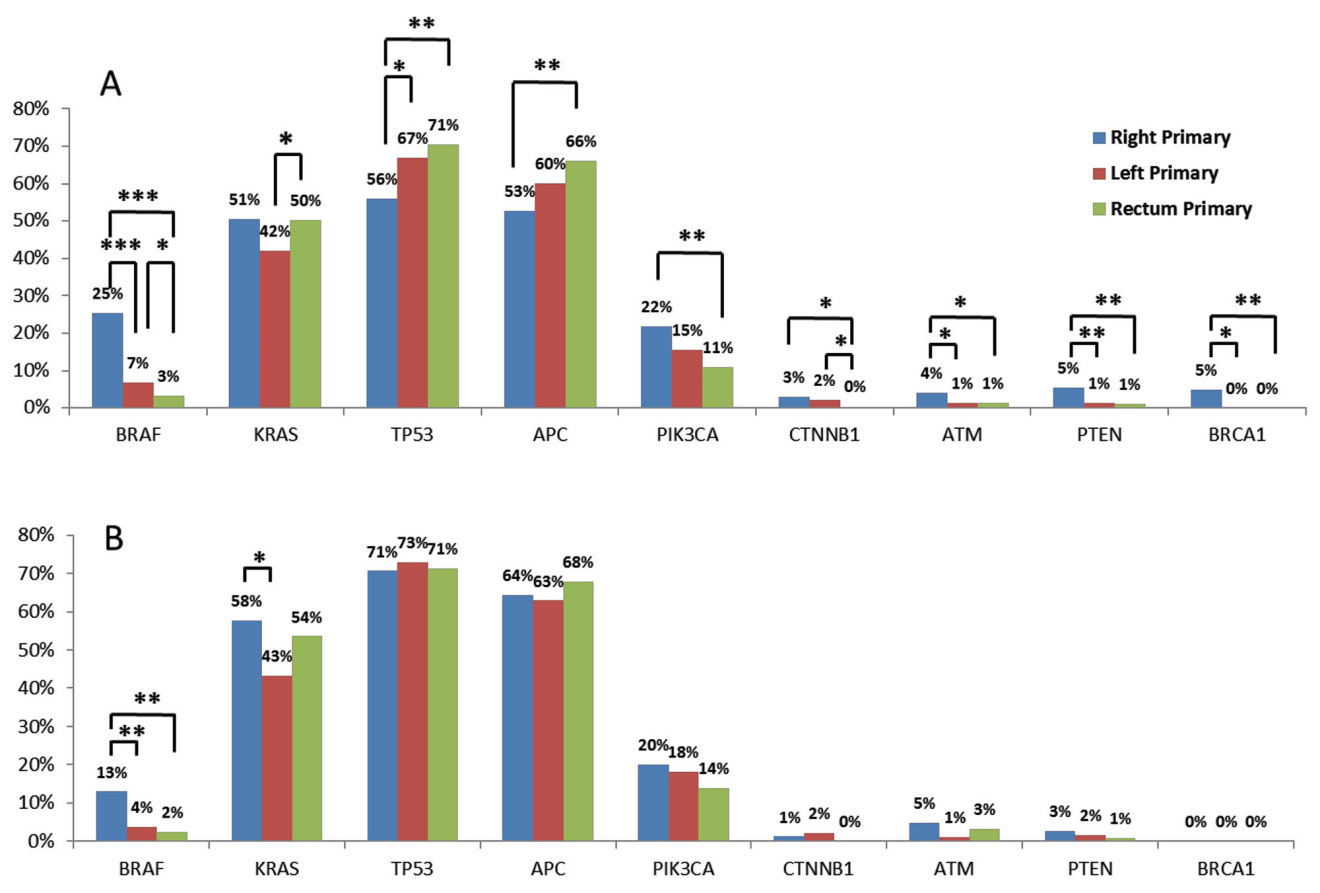
Gene mutation rates in primary tumors of right-sided colon tumors, left-sided colon tumors, and rectal tumors * = p < 0.05, ** = p < 0.01, *** = p < 0.001 by Chi-squared tests. **(A)** Comparison in all primary tumors; **(B)** comparison in MMRp tumors.

Rates of pathogenic and presumed pathogenic mutations were compared among right-sided colon, left-sided colon, and rectal tumors (Figure 2A). *BRAF* mutations were seen in 25% of right-sided colon, 7% of left-sided colon, and 3.2% of rectal tumors. The majority of *BRAF* mutations seen in right and left colon tumors were V600E mutations (42 out of 45 [93%], right; 17 out of 22 [77%], left), which was not the case for the rectum, where only half (5 out of 10 or 50%) were *BRAF* V600E mutations. On further comparison of right and left colon and rectal cancers, *TP53* and *APC* were significantly more frequently mutated in rectal than right colon tumors, whereas the converse was true for *PIK3CA*, *CTNNB1*, *ATM*, *PTEN*, and *BRCA1*. Notably, *BRAF*, *PIK3CA*, *CTNNB1*, *ATM*, and *PTEN* showed decreasing mutational frequencies from the right side to the left side of the colon to the rectum, while increasing mutation frequencies of *TP53* and *APC* were seen.

#### MMRd (mismatch repair deficient) tumors

**Figure 3:**
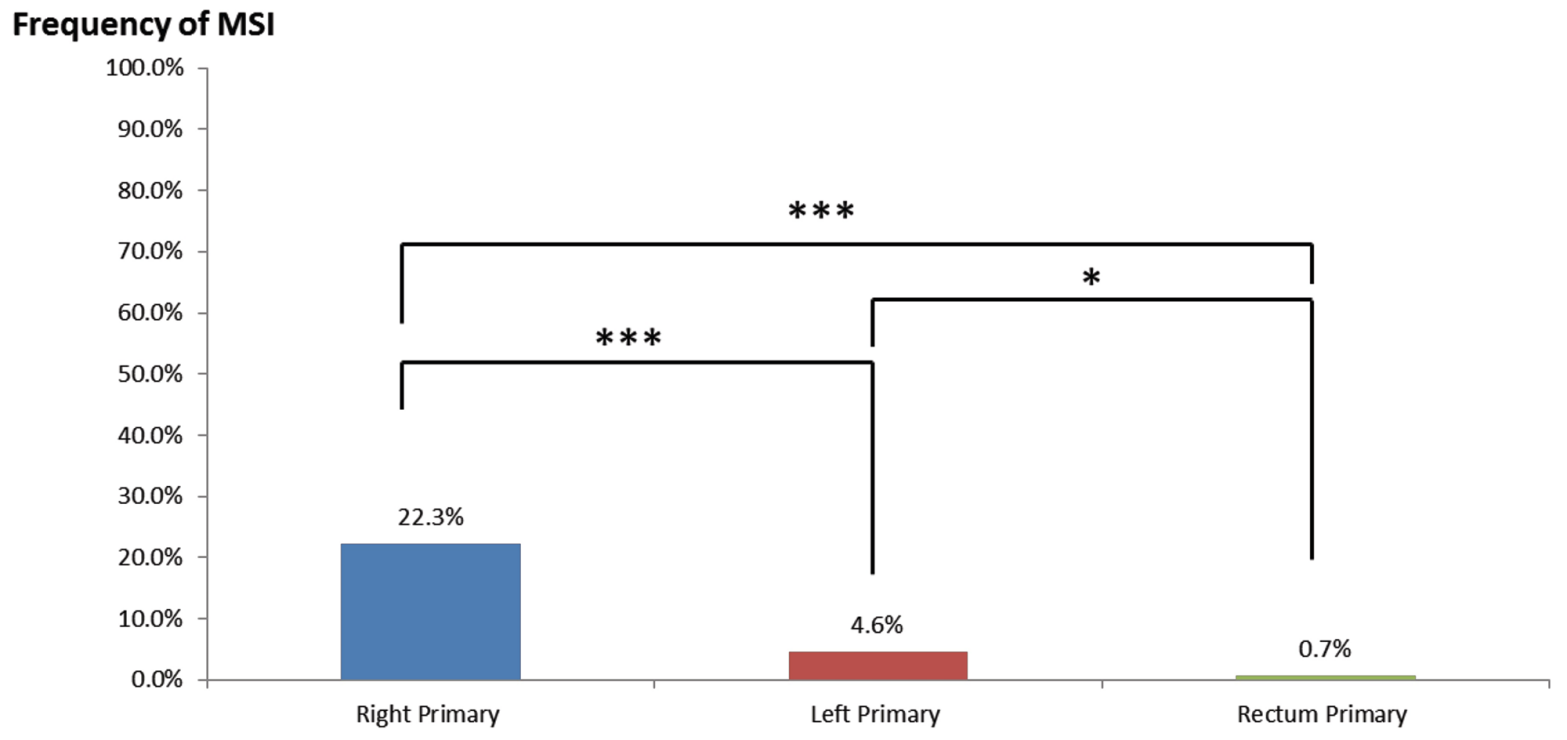
MMRd frequency in right-sided colon cancers, left-sided colon cancers, and rectal cancers * = p < 0.05, *** = p < 0.001 by Chi-squared tests.

In the portion of cases that underwent microsatellite instability fragment analysis (MIA), MMRd was seen in 22.3% (25/112) of right-sided colon tumors, 7.1% (3/42) of left-sided colon tumors, and only 0.7% (1/133) of rectal tumors—all were statistically significantly different from each other (Figure 3). Multivariate analysis by logistic regression confirmed that, after correction for gender, age, and tumor classification (primary vs. metastatic), all differences remained statistically significant (left vs. rectum odds ratio [OR] = 8.84, P = 0.039; right vs. left OR = 5.89, P = 0.0000037; right vs. rectum OR = 52.04, P = 0.00015).

#### MMRp (mismatch repair proficient) tumors

We also looked at the mutational landscape in the subgroup of MMRp tumors (Figure 2B), which incorporated 78% (87/112) of right-sided colon tumors, 95% (207/217) of left-sided colon tumors, and 99% (132/133) of rectal tumors. In concordance with *BRAF* mutation patterns seen in all primary MMRp tumors just described, the highest *BRAF* mutation rate was seen in the right colon (13%), whereas the lowest rate was in the rectum (2%, P = 0.003). In left-sided colon tumors, the *BRAF* mutation rate was 4%, which was significantly lower than right-sided colon tumors (P = 0.004). The *PIK3CA* and *PTEN* mutation rates in MMRp tumors decreased from right-sided to left-sided colon to rectum; however, the differences were not statistically significant.

#### Tumor mutational load (TML)

**Figure 4:**
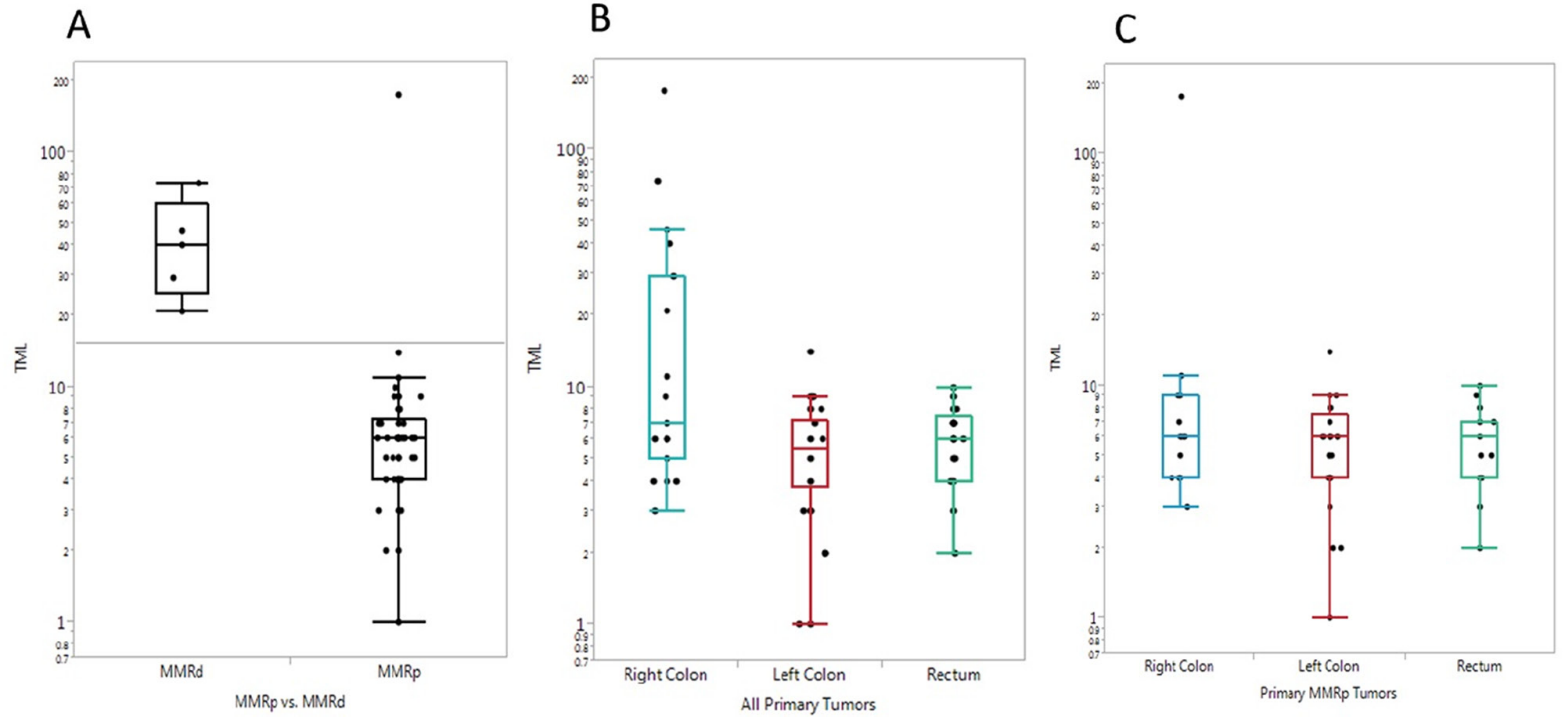
Box plots of mutational load seen in each tumor and correlation with MMR status and tumor location **(A)** Mutational load per tumor (number of mutations per megabase) in MMRp and MMRd tumors. **(B)** Mutational load comparison in primary tumors of the right colon, left colon, and rectum. **(C)** Mutational load comparison in MMRp primary tumors of the right colon, left colon, and rectum.

Mutational load was calculated for 70 primary colorectal tumors sequenced for 592 genes by NGS (Figure 4). Sixty-four tumors showed less than 17 mutations/megabase (MB), 57 of which had microsatellite instability (MSI), shown by fragment analysis, and all were MMRp. Six tumors had greater than 17 mutations/MB (range, 21 - 176 mutations/MB), and 83% of these tumors (5/6) were MMRd (P = 0.0183), while one was MMRp with a double *POLE* mutation (V411L and P697S, 246 total mutations).

Right-sided colon tumors had an average of 24.5 mutations/MB (standard error [SE] = 9.46), which was higher than that seen in left-sided colon tumors (5.5 mutations/MB, SE = 0.52, P = 0.0598) and rectal tumors (5.9 mutations/MB, SE = 0.457, P = 0.0647).

It is worth noting that out of the 70 tumors assessed for mutational load, 5 of 19 (26%) right-sided colon tumors, 0 of 29 (0%) left-sided colon tumors, and 0 of 15 (0%) rectal tumors were MMRd.

When mutational load was calculated for MMRp tumors only (n = 58), the average TML in right-sided colon was 18.3 mutations/MB and was 5.7 mutations/MB in both left-sided colon and rectal tumors. The differences were not significant and the numerical difference was caused by the one right-sided colon tumor with a *POLE* mutation.

#### Protein expression

**Figure 5:**
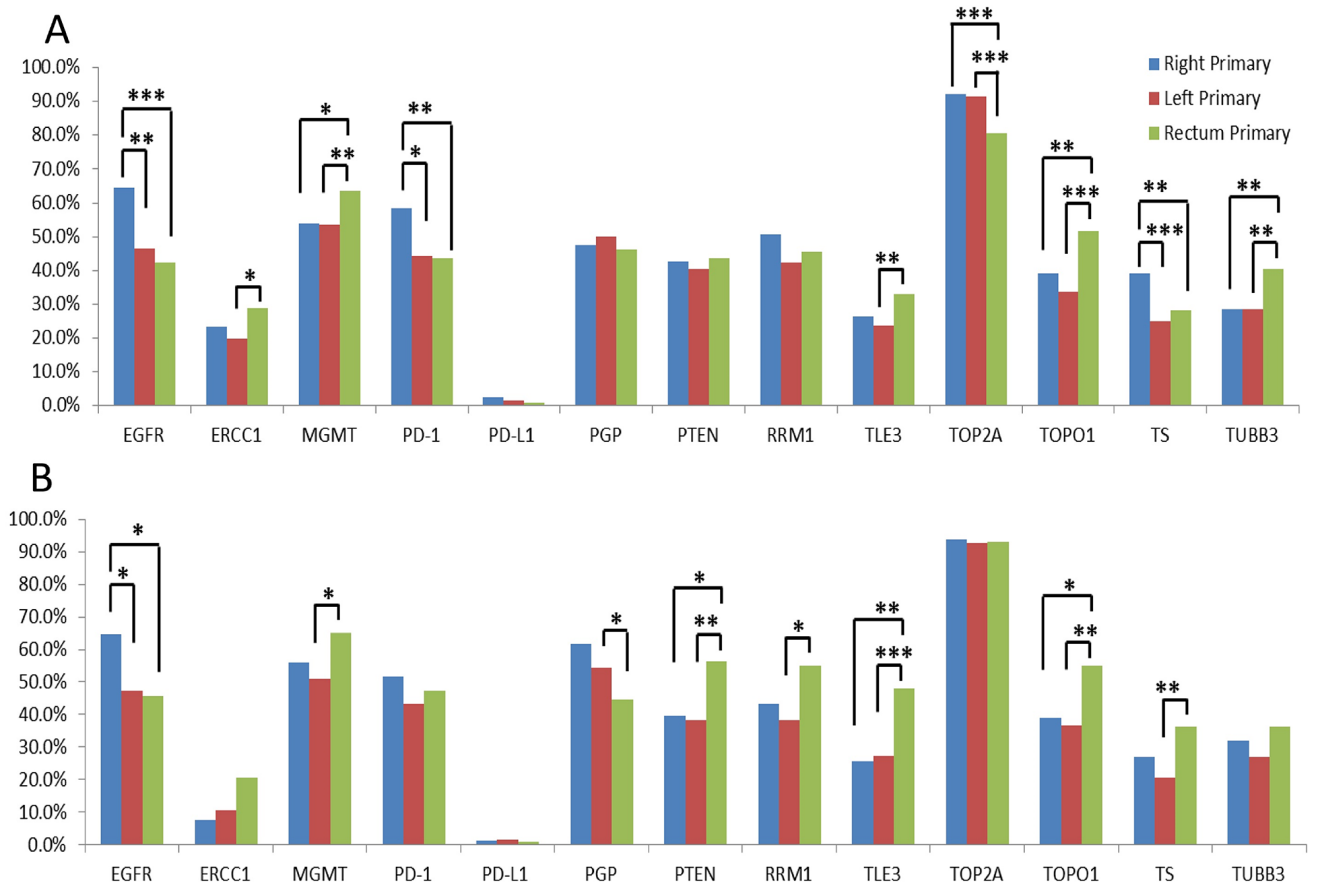
Protein expression rates in primary tumors of the right colon, left colon, and rectum * = p < 0.05, ** = p < 0.01, *** = p < 0.001 by Chi-squared tests. **(A)** Comparison in all primary tumors; **(B)** comparison in MMRp tumors.

As shown in Figure 5, IHC revealed statistically significant differences in protein expression of markers in right-sided, left-sided colon, and rectal tumors, either when all primary tumors (Figure 5A) or when only MMRp tumors were compared (Figure 5B). Protein expression of 12 markers (MGMT, TOPO1, TOP2A, ERCC1, TUBB3, EGFR, TS, PGP, PTEN, RRM1, TLE3 and PD-1) was significantly different in rectal tumors when compared to left- or right-sided colon tumors, or both. In particular, EGFR expression was significantly higher in the right-sided tumors than the left-sided and rectal tumors, independent of MMR status; in contrast, TOPO1 expression was higher in rectal tumors compared to the right-sided and left-sided colon, again independent of MMR status. In addition, PD-1 expression on tumor infiltrating lymphocytes was more often seen in right-sided tumors, but not when only MMRp tumors were evaluated, indicating that the difference was likely associated with MMR status.

**Figure 6:**
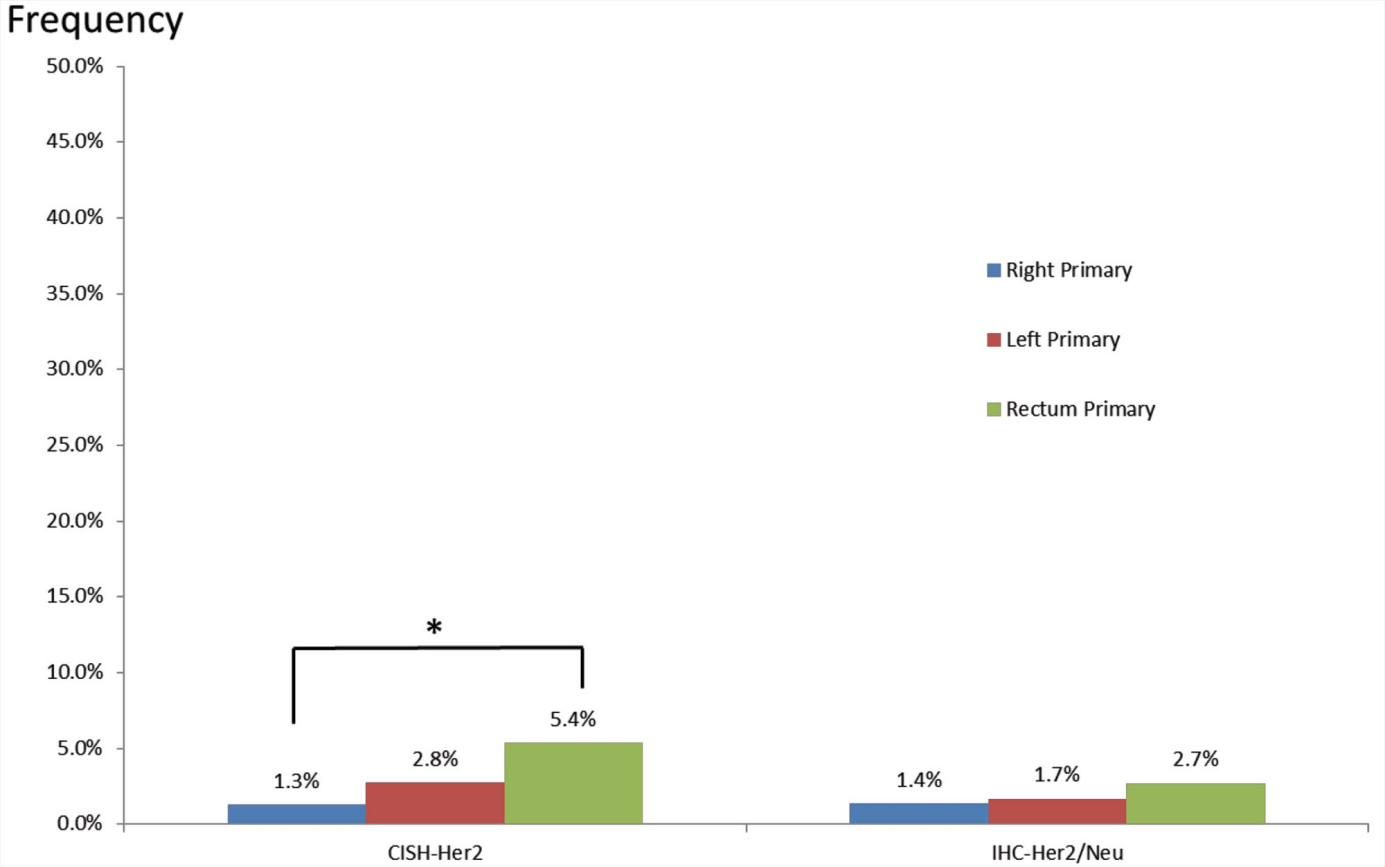
Her2/neu amplification and overexpression in primary tumors of the right colon, left colon, and rectum

Her2/neu gene amplification and protein expression were evaluated by chromogenic *in situ* hybridization (CISH) and IHC, respectively (Figure 6). Even though Her2/neu frequency was low overall, rectal tumors carried the highest rate of gene amplification and overexpression (5.4% and 2.7%, respectively) when compared to left-sided (2.8% and 1.7%) and right-sided (1.3% and 1.4%) colon tumors. Her2/neu gene amplification was significantly higher in rectal tumors compared to right-sided colon tumors (P = 0.03).

## DISCUSSION

These findings raise important questions: are right- and left-sided colon cancers biologically distinct from rectal cancers, and could this impact the choice of therapy for these distinct cancer groups [8]? Mutational analyses of primary tumors have suggested important differences in oncogenic driver mutations between colon and rectal cancers. In one study of 158 patients with metastatic colon cancers and 64 patients with metastatic rectal cancers, colon cancers had a higher *BRAF* mutation rate compared to rectal cancers (13.3% vs. 3.1%, P < 0.05), but lower rates of *NRAS* (0.6% vs. 12.5%, P < 0.05) and *TP53* mutations (17.7% vs. 29.7%, P < 0.05). *KRAS* mutation rates were similar (37.3% vs. 34.4%; P > 0.05) [9]. These differences offer insight into the potential use of EGFR inhibitors due to the fact that only patients with *RAS* and *BRAF* wild-type tumors derive benefit [10, 11] (although results from the study by Venook and colleagues suggested that cetuximab might actually be detrimental to patients with right-sided tumors, regardless of *RAS* status [3], meaning that other factors are coming into play).

Another study of 1,443 colon and rectal cancers demonstrated that a greater percentage (19-37%) of proximal colon cancers are MMRd compared to only 1.6% of rectal cancers, suggesting that rectal cancers may be less responsive to novel immunotherapies such as anti-PD-1 or anti-PD-L1 (checkpoint) inhibitors [12, 13].

Using a large cohort of colorectal tumors submitted for profiling, we performed a systematic comparison of molecular alterations between tumors collected from different locations, including a large cohort of 1,445 rectal tumors. This sample size is in sharp contrast to the previous work done by The Cancer Genome Atlas (TCGA) which compared only 132 colon and 62 rectal tumors, although the TCGA data was obtained prospectively [14]. Despite the exclusion of a large number of colon tumors from profiling analyses, specifically those without clear annotation of tumor location (N = 8,041) and those taken from the transverse colon (N = 116), ours is still one of the largest cohorts analyzed and reported to date. All tumors were microdissected and only tumor tissue was sampled for NGS, eliminating potential confounding mutations from non-malignant stromal regions that could influence genetic profiles by inappropriately suggesting epithelial-mesenchymal transition [15]. Our biomarker analysis included only primary tumors due to the significantly different molecular profiles of primary and metastatic CRC [16].

In our analysis, rectal tumors were more common in men whereas right-sided tumors were more common in women. This finding agrees with those from previous studies demonstrating that women are more likely to have right-sided tumors [17, 18]. The tendency of women to develop right-sided tumors likely reflects the protective role of estrogen in the development of CRC due to its anti-inflammatory effect on the tumor microenvironment [19]. In fact, estrogen receptor expression is higher in right-sided than left-sided colon tumors [20]. The rightward shift toward more proximal colon MMRd tumors in older female patients may be explained by the inevitable reduction in estrogen levels with age [17, 21].

We show a dramatic decrease in MMRd tumors when moving from right to left colon to rectum. MSI is caused by loss of MMR function and is generally found in about 15% of all CRCs, including around 3% of CRCs associated with hereditary non-polyposis CRC (HNPCC), also known as Lynch Syndrome [22]. Regardless of origin, patients with MMRd tumors are resistant to conventional chemotherapies, including 5-FU [23-25]. However, these tumors carry a hypermutated phenotype associated with the generation of neoantigens, making them promising candidates for immune checkpoint inhibition [13, 24]. Our results show that approximately one quarter of right-sided colon tumors are MMRd and may benefit from treatment with checkpoint inhibitors, providing a very promising therapeutic opportunity for this subgroup of colon tumors with poor prognosis and limited treatment options [3, 6, 26]. Others have observed that even in MMRp tumors, higher mutational load positively correlates with lymphocyte infiltration and improved CRC-specific survival [27].

We show a significant decrease in the *BRAF* mutation rate from right colon (25%) to left colon (15%) to rectum (3%). These values contrast significantly with the previously reported 5-10% *BRAF* mutation rate [28, 29], probably due to the fact that colon (both sides) and rectal tumors were grouped together in previous studies. Our findings confirm the results of previous studies demonstrating the right-sided predominance of *BRAF*-mutated CRC (comprising 22% of right-sided, 4% of left-sided, and 2% of rectal CRCs) [28]. Mutated *BRAF* has been associated with MSI and CIMP [30]. Patients with *BRAF*-mutated CRCs carry the worst overall prognosis among CRC patients and are notoriously refractory to therapy, despite best efforts with currently available *BRAF*-targeted agents and various combination therapies [31]. One analysis of three independent cohorts that grouped rectal tumors with left-sided colon tumors showed that, although *BRAF* mutations were more prevalent in right-sided tumors, right-sided location was a negative prognostic variable independent of *BRAF* mutational status [32]. Patients with *RAS* and *BRAF* wild-type right-sided tumors from the PRIME, PEAK, and 181 studies had inferior OS, progression-free survival (PFS), and objective response rate (ORR), and similar survival outcomes were observed in subgroup analyses of *RAS* wild-type tumors from the FIRE-3, CRYSTAL, and CALBG/SWOG 80405 studies [3, 5, 33]. Our observations suggests that within the left-sided group, there is a decrease of *BRAF* mutation rate from left colon to rectum and that the different *BRAF* mutation rates might contribute to the different behavior of left- and right-sided colon cancers and rectal cancers.

Additional important findings from our study include the significantly lower activation of the PI3K/AKT/mTOR pathway in rectal tumors compared to left- and right-sided colon tumors, with lower mutation rates of *PIK3CA* and *PTEN* in rectal tumors. Although agents that directly target the PI3K/AKT/mTOR pathway have been shown to be ineffective in CRC [34], *PIK3CA* mutations have been implicated in EGFR-targeted therapy resistance, as well as increased benefit from aspirin treatment [35-38]. Loss of *PTEN* expression may also confer resistance to EGFR-targeted therapy [39, 40]. The higher rate of activating *PIK3CA* mutations and *PTEN* loss in left- and right-sided colon cancers compared to rectal cancers could contribute to a lower response to anti-EGFR therapies in some colon cancers and supports the use of alternative treatments. Epigenetic modifications that confer sensitivity to EGFR-targeted therapy (e.g., demethylation of the *EREG* promoter) occur more commonly in rectal tumors, but such epigenetic modifications were not assessed in our study [41]. Additionally, the insulin-like growth factor (IGF) pathway has also been implicated in CRC oncogenesis but was not analyzed in our study [42]. In a study of 69 patients with *KRAS* exon 2 mutant CRC, IGF-1 expression was higher in rectal than colon tumors, approaching statistical significance (P = 0.06) and suggesting that rectal cancers harbor oncogenic pathways that are different from colon cancers [43]. These are certainly markers to consider for future confirmatory analyses.

Rectal cancers were shown to have higher expression of TOPO1 than colon cancers, which could be useful in the direction of irinotecan treatment—first demonstrated in the UK MRC FOCUS trial, but with limited validation from other trials [44]. In the MAVERICC study, which compared first-line treatment using mFOLFOX6/bevacizumab with FOLFIRI/bevacizumab in patients with mCRC, those with left-sided mCRC attained longer median PFS with FOLFIRI/bevacizumab than mFOLFOX6/bevacizumab (13.8 vs. 10.2 months, P = 0.040); however, this was not seen in patients with right-sided metastatic colon cancer [45]. Although this difference could be attributed to higher TOPO1 expression in the rectal tumors that were included in the left-sided cohort, further investigation is needed.

Xenograft model studies [46] and the multicenter phase II HERACLES trial [47] indicate that the Her2/neu pathway is an important therapeutic target in CRCs. Our current study suggests a trend toward higher prevalence of Her2/neu overexpression and amplification in rectal cancers compared to colon cancers, despite the low levels observed overall. The rate of Her2/neu overexpression in rectal cancer has previously been reported to be as high as 26.7% [48]. This finding could be helpful in the selection of patients for future clinical trials of Her2/neu-targeted therapies.

Limitations of our study include lack of DNA methylation evaluation, CMS classification, and the restriction to molecular diagnostic tests widely used in the clinical setting (IHC, ISH, and NGS). In addition, primary or metastatic tumor specimens were categorized according to site of procurement, with no patient identification. Therefore it is possible that the primary cohort may contain samples from primary sites in patients who also had metastatic tumors samples analyzed.

Many tumors were excluded because their primary site was unknown (N = 8,563), significantly limiting the power of this study to detect novel CRC mutations. Metastatic tumors (especially from the colon) were underrepresented relative to primary tumors, and patients with earlier stage colon cancer were also likely underrepresented in this study. This is because the stage at diagnosis was not reported and tumors are most commonly profiled at a later stage. Prior work by El-Deiry and colleagues demonstrated clear molecular differences among different metastatic sites of CRC and primary tumors, including higher Her2/neu expression in lung metastases than primary tumors (4% vs. 1.8%, P = 0.028), higher rates of *KRAS* mutations in brain and lung metastases than other sites (65% vs. 59% vs. 47%, P = 0.07 and < 0.01, respectively), and higher TOPO1 expression in metastatic tumors than primary tumors (52% vs. 30%) [16]. Rectal tumor specimens also may have received neoadjuvant chemoradiation, potentially altering their IHC profile. In one study of 225 resected rectal cancer specimens, Her2/neu amplification was seen in 45% of untreated tumors but only in 23% of tumors exposed to neoadjuvant chemoradiation (P = 0.009) [48]. Finally, our analysis of tumor mutational load is exploratory given the small cohort of tumors assessed (N = 70).

**Figure 7:**
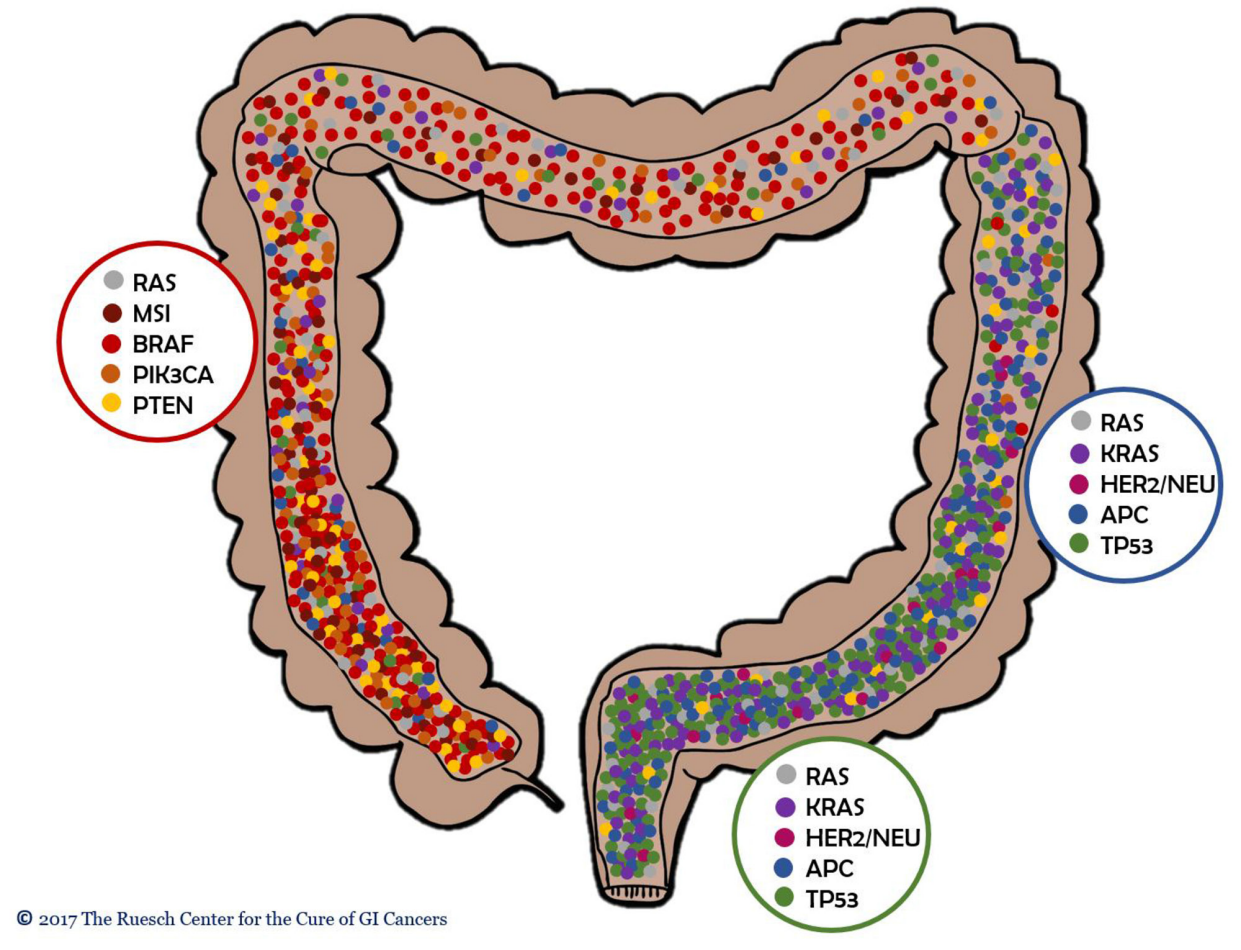
CRCs carry a continuum of molecular alterations from right to left, rather than having a sharp, clear-cut distinction

Tumor sidedness has been shown to have a profound influence on clinical outcomes and the current study demonstrates, not only that left- and right-sided colon cancers differ in tumor biology, but also that rectal cancer have a tumor biology that is distinct from colon cancers. Furthermore, it appears that CRCs carry a continuum of molecular alterations from right to left, rather than having a sharp, clear-cut distinction (Figure 7).

In conclusion, our cohorts of colorectal tumors have different rates of MMRd, *BRAF* and *PIK3CA* mutations, tumor mutational burden, and TOPO1 and Her2/neu expression, providing vital clinical information that has the potential to guide treatment selection for individual patients and change the design of future clinical trials to stratify patients by site of primary tumor.

## MATERIALS AND METHODS

### Data collection

colorectal tumors profiled by a CLIA-certified laboratory (Caris Life Sciences, Phoenix, AZ) between 2009 and 2016 were de-identified and retrospectively analyzed for molecular alterations. Tumor origins were taken from submitted pathology reports and confirmed by board certified pathologists. Tumor samples with origins annotated as “colon, NOS (not otherwise specified)” were excluded from the study; only those tumors originating from specified colon or rectal regions were included. Right-sided tumors were defined as arising from the cecum to the hepatic flexure and left-sided tumors from the splenic flexure to the sigmoid colon. Tumors of the transverse colon were deemed neither right- nor left-sided and were excluded from analysis, as was also the case for the retrospective CALGB/SWOG 80405 analysis [3]. Samples taken from confirmed tumor origins were considered primary tumors while samples taken from organs other than the primary were considered metastases.

### Multiplatform testing

Immunohistochemistry was performed on full formalin-fixed paraffin-embedded (FFPE) sections on glass slides. Slides were stained using an automated system (Benchmark, Ventana Medical Systems, Tucson, AZ; Autostainer, DAKO, Carpinteria, CA) as per the manufacturer’s instructions, and were optimized and validated per CLIA/CAO and ISO requirements. Tumor cells were scored for all proteins of interest with the exception of PD-1, which was scored on tumor infiltrating lymphocytes. Staining was scored for intensity (0 = no staining; 1+ = weak staining; 2+ = moderate staining; 3+ = strong staining) and staining percentage (0-100%). Results were categorized as positive or negative by defined thresholds specific to each marker, based on published clinical literature that associates biomarker status with patient responses to therapeutic agents. A board-certified pathologist evaluated all IHC results independently. Protein IHC details can be found in Supplementary Table 1.

MSI was tested with MIA. MIA included fluorescently labeled primers (Promega, Madison, WI) for co-amplification of seven markers including five mononucleotide repeat markers (BAT-25, BAT26, NR-21, NR24, and MONO-27) and two pentanucleotide repeat markers (Penta C and D). The mononucleotide markers were used for MSI determination while the pentanucleotide markers were used to detect either sample mix-ups or contamination. A tumor sample was considered MMRd if two or more mononucleotide repeats were abnormal. If one mononucleotide repeat was abnormal or repeats were identical between the tumor and adjacent normal tissue, then the tumor sample was considered MMRp.

CISH was used to detect Her2/neu gene amplification (INFORM HER-2 Dual ISH DNA Probe Cocktail, Ventana, Tucson, AZ). NGS was performed on genomic DNA isolated from FFPE tumor samples using either the MiSeq platform or the NextSeq platform (Illumina, Inc., San Diego, CA), and no matched normal tissue was sequenced. For tumors tested with MiSeq, specific regions of the genome were amplified using the Illumina TruSeq Amplicon Cancer Hotspot panel. For tumors tested with NextSeq, a custom-designed SureSelect XT assay was used to enrich 592 whole-gene targets (Agilent Technologies, Santa Clara, CA). All variants were detected with > 99% confidence based on allele frequency and amplicon coverage with an average sequencing depth of coverage of > 500 and with an analytic sensitivity of 5%. Tumor enrichment was achieved by harvesting targeted tissue by manual microdissection performed on all cases prior to molecular testing. Candidate slides were examined under a microscope and areas containing tumor cells were circled. A laboratory technician harvested targeted tissues for extraction from the marked areas using a dissection microscope. The areas marked and extracted were microscopically reexamined on post-microdissected slides and adequacy of microdissection was verified by a board certified pathologist.

Genetic variants identified were interpreted by board-certified molecular geneticists and categorized as ‘pathogenic,’ ‘presumed pathogenic,’ ‘variant of unknown significance,’ ‘presumed benign,’ or ‘benign,’ according to the American College of Medical Genetics and Genomics (ACMG) standards. When assessing mutation frequencies of individual genes, ‘pathogenic’ and ‘presumed pathogenic’ were counted as mutations while ‘benign’ and ‘presumed benign’ variants and ‘variants of unknown significance’ were excluded. TML was measured for tumors sequenced using the NextSeq platform (592 genes and 1.4 MB sequenced per tumor) by counting all non-synonymous missense mutations found per tumor that had not been previously described as germline alterations.

### Statistical analysis

Bivariate comparisons of biomarker profiles—protein expression (IHC), gene amplification (CISH), and gene mutations (Sanger, NGS)—between groups were performed using chi-squared tests (IBM SPSS Statistics, Version 23), and significance was defined as P < 0.05. All reported P-values were two-sided, and significance was defined as P < 0.05. A logistic regression model was used for multivariate analysis using gender, age, and primary versus metastatic tumor as covariates, which were entered directly. Only primary tumors were used for the analysis. Metastatic tumors were investigated separately and data are not shown to avoid confusion. Focusing exclusively on primary tumors allows for a more homogenous population of tumors.

## SUPPLEMENTARY MATERIALS TABLE



## Abbreviations

ACMG: American College of Medical Genetics and Genomics
CI: confidence interval
CIMP: CpG island methylator phenotype
CISH: chromogenic *in situ* hybridization
CMS: consensus molecular subtype
CRC: colorectal cancer
FFPE: formalin-fixed paraffin-embedded
HNPCC: hereditary non-polyposis colorectal cancer
HR: hazard ratio
IGF: insulin-like growth factor
IHC: immunohistochemistry
MB: megabase
mCRC: metastatic colorectal cancer
MIA: microsatellite instability fragment analysis
MMRd: mismatch repair deficient
MMRp: mismatch repair proficient
mOS: median overall survival
MSI: microsatellite instability
NGS: next-generation sequencing
OR: odds ratio
SE: standard error
TCGA: The Cancer Genome Atlas
