# Supplementary material for: Comparative molecular analyses of left-sided colon, right-sided colon, and rectal cancers
Source: Oncotarget. 2017 Sep 21;8(49):86356–68. doi: 10.18632/oncotarget.21169 (PMC5689690; doi:10.18632/oncotarget.21169)
Supplement: Supplementary file 1 [file oncotarget-08-86356-s001.pdf]

Comparative molecular analyses of left-sided colon, right-sided colon, and rectal cancers

SUPPLEMENTARY MATERIALS

Supplementary Table 1: Protein immunohistochemistry details

Immunohistochemistry Biomarker	Antibody	Manufactor	Location
cMET	SP44	Ventana	Tucson, AZ
EGFR	31G7	Zymed/Invitrogen	Frederick, MD
ERCC1	8F1	ABCAM	Cambridge, MA
Her2	4B5	Ventana	Tucson, AZ
MGMT	MT23.2	Invitrogen	Frederick, MD
PD-1	NAT105	Ventana	Tucson, AZ
PD-L1	SP142	Spring Bio	Pleasanton, CA
PGP	C494	Invitrogen	Frederick, MD
PTEN	6H2.1	Dako	Carpinteria, CA
RRM1	polyclonal	Proteintech	Rosemont, IL
TLE3	POLY	Lifespan	Seattle, WA
TOP2A	3F6	Leica	UK
TOPO1	1D6	Leica	UK
TS	TS106/4H4B1	Invitrogen	Frederick, MD
TUBB3	(POLY)	Covance	Princeton, NJ
